# A Retrospective Cohort Analysis Comparing Analytic and Holistic Marking Rubrics in Medical Research Education

**DOI:** 10.1177/23821205241277337

**Published:** 2024-08-28

**Authors:** Siew Wan Yeo, Christina Signorelli, Khanh Vo, Greg Smith

**Affiliations:** 1 UNSW Medicine and Health, UNSW Sydney, Kensington, Australia; 2 Kids Cancer Centre, Sydney Children's Hospital, Randwick, Australia

**Keywords:** rubric, consistency, holistic marking, analytic rubrics, mark differences, adjudication, inter-rater reliability

## Abstract

**OBJECTIVES:**

The use of analytic rubrics in assessing and grading students’ performance has become more prominent among instructors due to its reliability and validity in ensuring consistency in student evaluation. However, there is limited evidence demonstrating the consistency of examiner judgments between analytic marking rubrics and holistic marking rubrics.

**METHODS:**

Therefore, we aimed to compare the consistency of marks given using holistic marking methods and analytic rubrics at an Australian university by analyzing the mean mark differences and number of adjudications between two rubric types as well as the inter-rater reliability between two assessors. We analyzed all scores for project manuscripts between 2016 and 2021 for Honours medical students. We compared the mean mark differences graded using the Kruskal–Wallis test and Welch *t*-test. We used chi-squared tests to compare the frequency of adjudications for each rubric type. We assessed interrater reliability by comparing the marks between the two examiners utilizing Pearson correlation.

**RESULTS:**

We found that analytic rubrics have lower mean mark differences and fewer adjudicators are required. We showed a strong positive association between the consistency of marks given and the use of analytic rubrics when compared to holistic marking. Pearson correlation showed a low but stronger correlation between marks awarded by the two assessors when analytic rubrics were used (*r* = 0.36), compared to holistic marking rubrics (*r* = 0.24).

**CONCLUSIONS:**

Our findings suggest that the use of analytic rubrics may increase the consistency and reliability between two independent examiners in marking medical students’ work.

## Introduction

Marking rubrics guide assessors by emphasizing specific criteria, minimizing subjective judgment, and improving grading consistency.^1,2^ Rubrics can also improve the teaching and learning process.^1,2^ Thus, for effective evaluation of student performance, rubrics need to be reliable and valid.^3^ Interrater reliability refers to the grading consistency among different examiners for a particular variable.^4,5^ Rubrics can be designed in various ways: holistic or analytical, and generic or task-specific.^6^ Holistic rubrics provide a single overall score by considering all criteria simultaneously which simplifies the evaluation process.^7^ Analytical rubrics require the marker to assess each criterion independently, allowing for a more detailed evaluation and feedback, although this approach may be less suitable for tasks where it is challenging to separate individual criteria for marking.^8^ Generic rubrics are not task-specific; they are designed to be applicable across a range of tasks and contexts. Unlike task-specific rubrics, generic rubrics provide a more standardized approach that can be applied broadly, enhancing consistency in grading.

Australian medical honors students at the University of New South Wales (UNSW) are assessed using a holistic marking method (Supplemental Table 1), that is applied generically (ie, used across a range of tasks/contexts) and analytic rubrics (Supplemental Table 2), assessing students based on specific criteria in different domains. In terms of scoring, in Supplemental Table 1, the rubric assigns scores to several general criteria whereas in Supplemental Table 2, the rubric considers analytical indicators related to the factors of such evaluations, listing the factors underpinning each criterion. Both types of rubrics have been developed by a committee and based on the scholarly literature,^8,9^ as well as measurable learning outcomes set for the course which are linked to specific student outcomes that they are expected to achieve during their research year and in light of specific assessment types (eg, oral presentations and research reports).

The use of an analytic rubric in assessing student performance has become popular among instructors due to its reliability and validity in ensuring consistency in the evaluation process.^7,8^ Yet, there is a lack of research comparing the two types of rubrics in medical education settings, regarding mark consistency and particularly the need for adjudication.^10^ In addition, most existing research focuses on one-off assessments, rather than longitudinal use comparing the consistency of marks over time for both rubric types. While it is generally accepted that holistic rubrics are faster to use, there also remains a lack of quantitative data on the time savings in medical education settings compared to analytic rubrics. Addressing these gaps in the literature would provide valuable insights for medical educators in designing and implementing the most effective rubric type for their specific assessment needs, and to reduce the need for additional resources for inconsistent marking through the use of an additional adjudicator. Therefore, this research aims to compare the consistency of marks using holistic and analytic marking systems, hypothesizing that analytic rubrics will improve score consistency in medical research education and reduce the need for adjudication. This hypothesis will be tested with the following aims:

1. To compare the discrepancy between examiner pairs using analytic and holistic marking rubrics.
2. To assess the correlation of marks allocated by examiners using each rubric type and the need for adjudicators.

## Methodology

### Study design and participants

The study design was a retrospective cohort analysis. We had ethics approval from UNSW Human Research Ethics, which included a waiver of consent for this low-risk project. We collected the scores of all fourth-year medical students from UNSW doing an Honours degree, which comprises conducting a research project and producing a project manuscript. These scores were based on project manuscripts from 2016 to 2021. All students from these cohorts were included in the analysis and there were no exclusion criteria applied. At UNSW, student manuscripts were marked using a holistic rubric in the years 2016 to 2018 and from 2019 to 2021 using an analytic rubric. Two examiners provided scores, and if their scores differed significantly (ie, a difference of more than 10), an adjudicator assigned a final score in these situations.

### Statistical analysis

We calculated the mean marks of project manuscripts over three years of marking using holistic and analytic rubrics. To determine the consistency, we assessed the score difference between the two examiners (ie, the discrepancy) and compared the score difference between the two rubric types using a Kruskal–Wallis test between the years. We compared the frequency of adjudications required for each rubric type in the event of large score differences using chi-square analysis. We report descriptive statistics including proportions (*n* = number, %) and mean ± standard deviations where appropriate. We measured interrater reliability by comparing the marks between the two examiners on each rubric type using Pearson correlations. We analyzed data using the “RStudio Version 12.0-353” software.

## Results

We examined 325 student project manuscript marks, of which 234 were marked using analytic rubrics and 91 were marked using holistic marking rubrics. We observed a significantly lower mean score difference for project manuscripts when markers used analytic rubrics (mean = 3.46, SD = 2.87), compared to holistic marking (mean = 6.63, SD = 5.12, H(5) = (40.2), *p* < .05; Table 1).

**Table 1.** Project manuscript marks by holistic and analytic marking rubrics.

RUBRIC TYPE	YEAR	MEAN	SD	MEDIAN	MIN	MAX	*N*
Holistic rubric	2016	8.04	5.28	7.35	0.95	21.30	31
	2017	6.21	5.10	5.43	0.50	26.75	30
	2018	5.60	4.79	4.50	0.05	18.25	30
	Total	6.63	5.12	5.5	0.05	21.3	91
Analytic rubric	2019	5.04	5.17	4.0	0.00	24.00	23
	2020	3.45	2.85	2.50	0.00	9.00	31
	2021	3.26	2.39	3.0	0.00	12.00	180
	Total	3.46	2.87	3.0	0.00	24.00	234
Total		5.04	3.90	3.5	0.00	26.75	325

Overall, the number of adjudicators across 6 years was 33 (20% of all research projects examined; Table 2). Holistic marking methods required 2.75 times more adjudicators (*n* = 17, 18.7%) compared to analytic rubrics (*n* = 16, 6.8%), which was statistically significantly different (χ^2 ^= 10.1, *p* < .05).

**Table 2.** A Comparison of the Number of Adjudications Required for the Project Manuscripts by Marking Rubric Type.

RUBRIC TYPE	YEAR	TOTAL ADJUDICATIONS		*X*^2^ STATISTIC (DF)	*P*-VALUE
		*n*	%		
Holistic rubric	2016	8	10.1	10.1 (1)	<.05
	2017	3	10.0		
	2018	6	20.0		
	Total	17	18.7		
Analytic rubric	2019	4	17.4		
	2020	0	0		
	2021	12	6.7		
	Total	16	6.8		
Total		33	20.0		

Pearson correlation revealed a significant low positive correlation between the marks between the two examiners on both holistic (*r* = 0.24) and analytic rubrics (*r* = 0.36).

## Discussion

Our study comparing analytic and holistic marking rubrics in the context of medical research education found that analytic rubrics tend to produce more consistent grades, demonstrated by significantly lower average score differences for project manuscript marks when using analytic rubrics, and significantly fewer instances requiring adjudication. When analytic rubrics were used, the number of adjudicators needed approximately almost tripled in our study compared to analytic rubrics (Table 2). Recent literature suggests that using holistic and analytic rubrics in clinical performance assessments in conjunction with task-specific checklists, may produce more efficient evaluation.^10^ Yet, Reid et al^11^ demonstrated that generic rubrics resulted in fewer adjudications (*n* = 32, 10.22% for the first year and *n* = 27, 8.36% for the second year) compared to task-specific rubrics (*n* = 113, 35.53% for the first year and *n* = 80, 24.77% for the second year). Further research on the effectiveness of combining elements of holistic and analytic rubrics in medical education, and considering the “specificity” of tasks (ie, generic vs task-specific rubrics) could potentially address the limitations of each type. Achieving increased consistency between marks reduces the need for adjudicators, saving resources and marker time.

In this study, Pearson analysis revealed a stronger positive and linear correlation between markers when using analytic rubrics, indicating improved grading consistency as UNSW transitioned from holistic marking to analytic rubrics. However, the correlation remains low, suggesting lower inter-rater reliability or levels of agreement between two examiners. Some studies have suggested that even when using analytic rubrics examiner's actual processing may be more holistic, and examiners may use the criteria in a post hoc manner to justify decisions already made, possibly explaining low inter-rater reliability.^7,12^ Thus, reviewing rubrics may be necessary to minimize variation in interpretation and further improve the rubric's quality and applicability.

A strength of our study was that multiple data analysis methods were utilized to determine consistency supporting the notion that analytic rubrics provide a consistent marking in written and assessments, as compared to holistic marking. However, the sample size from 2016 to 2021 was modest, which may have resulted in a lower number of adjudicators and limited statistical power for our analyses. There may also be other unaccounted factors influencing marking consistency, particularly as the rubrics were across different years. The findings are from a single institution and may not be generalizable to other student populations or academic disciplines outside of medicine, potentially limiting the external validity of the study.

## Conclusions

Overall, our study suggests that analytic rubrics yield greater consistency in marking compared to holistic marking, supported by lower mean mark differences between examiners, reduced adjudication needs, and a significant positive correlation between examiners. These findings are valuable for developing high-quality rubrics to enhance the accuracy of feedback provided to medical students involved in research. While our study has its strengths, further studies are necessary to validate these findings.

## Supplemental Material

sj-docx-1-mde-10.1177_23821205241277337 - Supplemental material for A Retrospective Cohort Analysis Comparing Analytic and Holistic Marking Rubrics in Medical Research Education Supplemental material, sj-docx-1-mde-10.1177_23821205241277337 for A Retrospective Cohort Analysis Comparing Analytic and Holistic Marking Rubrics in Medical Research Education by Siew Wan Yeo, Christina Signorelli, Khanh Vo and Greg Smith in Journal of Medical Education and Curricular Development

sj-docx-2-mde-10.1177_23821205241277337 - Supplemental material for A Retrospective Cohort Analysis Comparing Analytic and Holistic Marking Rubrics in Medical Research Education Supplemental material, sj-docx-2-mde-10.1177_23821205241277337 for A Retrospective Cohort Analysis Comparing Analytic and Holistic Marking Rubrics in Medical Research Education by Siew Wan Yeo, Christina Signorelli, Khanh Vo and Greg Smith in Journal of Medical Education and Curricular Development
