# Supplementary material for: A Retrospective Cohort Analysis Comparing Analytic and Holistic Marking Rubrics in Medical Research Education
Source: J Med Educ Curric Dev. 2024 Aug 28;11:23821205241277337. doi: 10.1177/23821205241277337 (PMC11359436; doi:10.1177/23821205241277337)
Supplement: sj-docx-1-mde-10.1177_23821205241277337 - Supplemental material for A Retrospective Cohort Analysis Comparing Analytic and Holistic Marking Rubrics in Medical Research Education [file sj-docx-1-mde-10.1177_23821205241277337.docx]

**Supplementary Materials for *“A comparison of generic rubrics and holistic marking in medical research education”***

**Supplementary Table 1: Holistic marking rubric used for marking honours project manuscript.**

| 50.0 – 64: | Pass (Honours Class III) |
| --- | --- |
| 65 – 74: | Credit (Honours Class II, Division 2) |
| 75 – 84: | Distinction (Honours Class II, Division 1) |
| 85 – 100: | High Distinction (Honours Class I) |
| 1. ABSTRACT, INTRODUCITON, AIMS AND HYPOTHESES   (worth 15% of overall mark) | |
| 1. Aims and outcome summarised succinctly and accurately in the Abstract. 2. Appropriate scientific background provided in the Introduction. 3. Limitations of the literature and areas of controversy identified in the Introduction. 4. Clear and valid aims and hypotheses or a research question stated. | |
| Grade : __________ Marks out of 100 | |
| 1. MATERIALS AND METHODS (worth 15% of overall mark) | |
| 1. Clearly described and/or fully referenced. 2. Appropriate and valid for the stated aims | |
| Grade : __________ Marks out of 100 | |
| 1. RESULTS (worth 20% of overall mark) | |
| 1. Represents an adequate body of work. 2. Sufficient controls and replicates performed if the project is experimental in nature. 3. Appropriate statistical and/or qualitative analyses performed. 4. Presentation of Results (Figures, Tables, etc) clear and logical. | |
| Grade : __________ Marks out of 100 | |
| 1. DISCUSSION (worth 45% of overall mark) | |
| 1. Relevant to the Introduction, Methods, and Results. 2. Logical in presentation and scientific content. 3. Shows critical/creative analysis. 4. Places findings in the context of past studies and has suggestions for future studies. | |
| Grade : __________ Marks out of 100 | |
| 1. REFERENCES (worth 5% of overall mark) | |
| 1. Citation style appropriate and consistent. 2. Citation list free of careless errors. | |
| Grade : __________ Marks out of 100 | |
| 1. OVERALL GRADE | |
| The final grade will be calculated with the following algorithm  Score at (A x 0.15) + (B x 0.15) + (C x 0.2) + (D x 0.45) + (E x 0.05) = Final Mark out of 100 | |
| Overall grade : __________ Marks out of 100 | |
| FURTHER COMMENTS | |
| Please provide comments regarding the student’s report. | |
| **(Expandable field-box)** | |

**Supplementary Table 2: Sample analytic rubric used for marking honours project manuscript.**

| **Criteria** | **Project Manuscript (if 9 is given for all criteria, full marks will be given i.e. 100%)** | | | | |
| --- | --- | --- | --- | --- | --- |
|  | **Fail (3)** | **Pass (5)** | **Credit (7)** | **Distinction (8)** | **High Distinction (9)** |
| **Abstract (x0.5)** | Significant inaccuracies in the summary of project | some inaccuracies in the summary of project | Fair summary of project, some aspect missing, and/or some error(s)/ Potentially inconsistent with main text | Good summary of project rationale, results and/or relevance | Concise, accurate and informative summary of project rationale, results and relevance |
| **Introduction, Research Aim(s) and Hypotheses or Research Questions (x1.0)** | Lacking details of the rationale of the project and scientific background. Factual errors or omissions in text. No links between research aim(s) and hypotheses or research questions and literature | Some introduction to the scientific background and the rationale of the project. More detail needed. Factual errors or omissions in text. Some links between research aim(s) and hypotheses or research questions and literature. | An adequate introduction of the scientific background and the rationale of the project. Some factual errors or omissions. Some links between research aim(s) and hypotheses or research questions and literature. | Good account of the scientific background and the rationale of the project. Clear links between research aim(s) and hypotheses or research questions and literature. Some minor errors. | Comprehensive, clear and concise account of the scientific background and the rationale of the project. Very clear links between research aim(s) and hypotheses or research questions and literature. |
| **Methodology (x1.0)** | Description of study design and analysis absent or unclear. | Description of study design and analysis lacking major details. Major errors in methodology. | Description of study design and analysis lacking major details. Minor errors in methodology. | Good description of study design and analysis, with minor errors. | Comprehensive, clear and detailed description of study design and analysis. |
| **Results, Description & Context (x1.5)** | No description of the experimental results given.  Lack of controls and replicates. Lack of appropriate data analysis (including statistics if appropriate) performed. Represents an inadequate body of work. | Description of the experimental results lacks required detail. Some conclusions or interpretation of results presented.  Insufficient controls and replicates used. Major errors or omissions in data analysis. Represents an inadequate body of work. | Description of the experimental results lacks required detail and appropriate reference to figures and tables (if appropriate). Some conclusions or interpretation of results presented.  Enough controls and replicates. Inappropriate data analysis (including statistics if appropriate) used in some parts. Represents an adequate body of work. | Clear description of the results with reference to tables and figures (if appropriate). No conclusions or interpretation of results presented.  Enough controls and replicates with minor miscalculations in analysis (including statistics if appropriate). Represents a large body of work. | Logical and clear description of the results with reference to tables and figures (if appropriate). No conclusions or interpretation of results presented.  Adequate sample size with appropriate data analysis (including statistics if appropriate) performed correctly. Represents an extensive body of work. |
| **Results: Presentation (x1.5)** | Results poorly presented or missing. Graph axes not labelled, and units of measurement absent (if appropriate). Legends do not explain the figures and/or tables in sufficient detail that they can be understood without reference to the text (if appropriate). Tables are not self-explanatory and/or are poorly labelled.  Major errors in data presentation. | Results are poorly presented. Most graph axes not labelled or missing units of measurement. Most legends do not explain the figures and/or tables in sufficient detail that they can be understood without reference to the text (if appropriate). Most tables are not self-explanatory and/or are poorly labelled.  Major errors in data presentation. | Results presented. Most graph axes labelled, and units of measurement given in parentheses. Not all legends explain the figures and/or tables in sufficient detail that they can be understood without reference to the text (if appropriate). Most tables are self-explanatory, some errors in description or labels.  Some significant errors in data presentation. | Results adequately presented. Graph axes labelled, and units of measurement given in parentheses. Not all legends explain the figures and/or tables in sufficient detail (if appropriate) that they can be understood without reference to the text. Most tables clearly labelled with footnotes and self-explanatory.  Some minor errors in data presentation. | Results very clearly presented. Graph axes labelled, and units of measurement given in parentheses. Legends explain the figures and/or tables in sufficient detail that they can be understood without reference to the text. Tables clearly labelled with clear footnotes and self-explanatory.  No errors in presentation. |
| **Discussion (x3.0)** | Results are restated with no interpretation or reference to previous scientific studies. Findings not placed within the broader context of the field.  No critical analysis of strengths and limitations of experiments. No future directions identified.  Little understanding of most major concepts. | Results are restated with little interpretation or reference to previous scientific studies. Major findings not placed within the broader context of the field.  No critical analysis of strengths and limitations of experiments. No future directions identified.  Misunderstanding of some major concepts. | Discussion is unclear in many areas. Some inappropriate interpretation of the results. Lacking reference to previous scientific studies. Significance of findings not placed within the broader context of the field.  Lacking some critical analysis of strengths and limitations of experiments. Future directions identified. | Discussion is clear. Appropriate interpretation of results, some reference to previous studies, but not always. Significance of findings placed within the broader context of the field.  Critical analysis of strengths and limitations of experiments. Future directions identified and justified. | Discussion is insightful, clear and logical. Extensive interpretation of the results with reference to previous scientific studies. Significance of findings extensively placed within the broader context of the field.  Comprehensive critical analysis of strengths and limitations of experiments. Future directions identified and clearly justified. |
| **References (0.5)** | Use of literature limited to a few articles and reviews. Poor attempt to explore literature. Many references inconsistent between text and list. Many major errors. | Significant over reliance on reviews or texts. Limited number of recent or seminal articles used. Many references inconsistent between text and list. Some major errors. Inappropriate citation style used. | Some over reliance on reviews or texts. Many articles not from recent or seminal publications. Many references inconsistent between text and list with many minor errors. Citation style incorrect/ inconsistent. | Predominant use of primary articles. Could have used more articles from recent or seminal publications. Citation style consistent. Reference list complete, but with some minor errors. | Predominant and comprehensive use of primary articles. Many articles presented from recent or seminal publications. Citation style correct and consistent throughout. Reference list completely accurate with no errors. |
| **Overall Presentation (x1.0)** | Major grammatical and spelling errors. Language used not professional. Numerous errors in figures and/or tables, or largely irrelevant (if appropriate). | Major grammatical and spelling errors. Professional expression used. Numerous errors in figures and/or tables, or largely irrelevant (if appropriate). | Some grammatical and spelling errors. Professional expression used. Most figures and/or tables accurate, but not so relevant (if appropriate). | A few grammatical and spelling errors. Professional expression and style used. All figures and/or tables accurate, focused and informative (if appropriate). | No grammatical or spelling errors. Professional expression and style used consistently. All figures and/or tables accurate, focused and informative (if appropriate). |
